# Contagium Animatum

**Published:** 1883-06

**Authors:** Frederick Peterson


### Contagium Animatum.

By Frederick Peterson, M. D.

We have a number of diseases which have the following peculiarities: They are general systemic diseases, that is, their effects, though partly local, are manifested upon the whole body of the individual. They are peculiar in that they are infectious or contagious, or both. They are communicable from one person to another. By touching an individual affected, by breathing the air he breathes, by touching anything he has worn or touched, even by passing his house, one is attacked by an insidious and virulent disease. To this class are to be reckoned scarlatina, small-pox, diphtheria, measles, septicæmia, cholera, yellow fever and other such specific, infectious diseases. Intermittent fever, though not contagious, is one of this class. All these form an entirely distinct group, differing in every respect from any of our other diseases. There is a sharp boundary line between them and such diseases, for instance, as rheumatism caused by exposure to cold and the common inflammations, such as pleurisy, nephritis, or hepatitis. They have nothing whatever in common.

Now let us look for a moment at the etiology of this strange class of specific, infectious diseases, which have puzzled us for ages.

These diseases must have some cause. That cause has been laid to changes in the atmosphere, of temperature, density or quantity of moisture. But many of these diseases prevail at any season, cold or warm, in any climate, in any density of atmosphere, near the level of the sea or on high mountains, in low, damp countries or in regions high and dry.

So the condition of the atmosphere has little to do with it. It has been supposed that poisonous gases in the atmosphere, arising from the earth or from decomposing organic substances, might cause these diseases. But no traces of such gases have been found, and none of the poisonous gases we know resemble in any particular such a gas as is suspected of being a cause of these specific, infectious fevers. But there is another factor to be looked at carefully when reasoning with regard to the cause of these diseases. Let us take septicæmia as an instance. You put a needle into your finger when sewing up a body after an autopsy. At this minute point, hardly perceptible on your finger, has something—call it a poison if you will—been introduced, which gradually extends from there, infects your whole system in a few hours or days, raises your temperature to 103–4–6°, your pulse to 120–40–60. Abscesses may form in your axilla, in all your joints, fatty degeneration of the heart may take place, inflammation of lungs, liver, kidneys, and of all the serous membranes, and then death occurs. But now a minute drop of your blood introduced into another will cause that other individual to undergo the same fatal septicæmia, and from him can another be inoculated in the same way, and so on through twenty-five different individuals, and still the virulence of the poison is as great as ever, and one minute drop of blood from the twenty-fifth individual is capable of destroying still others. This is not a matter of supposition, but of actual experiment in lower animals. How does this compare with the action of any of our poisons?—take even our strongest, say wourara or strychnia? To be sure, a certain, fixed and minute quantity of these will produce rapidly a fatal termination, but it still remains the same quantity of poison, and any of the blood of that person will not poison another, except in the proportion of its dilution. For instance, if ½ gr. of strychnia kills a man on being absorbed into the circulation, it remains a ½ gr. but diluted by some sixteen pints of blood. Hence a drop of that blood is entirely incapable of producing a fatal result in another. This brings out the distinctive point. The poison of the septicæmia is altogether different, for it has the faculty of increasing and not being diluted. In other words the poison, whatever kind it is—in septicæmia, in diphtheria, in small-pox, in scarlet fever, in cholera—*grows.* That is the chief point to remember, the characteristic that *this poison grows.* We know of nothing *in*-organic which has this power of increasing. It is something having life, organic, self-propagating. Hence the theory known as the "Germ Theory," which supposes these specific, infectious diseases caused by inferior organisms which find entrance to the blood, through wounds, through the alimentary canal, through the delicate pulmonary mucous membrane, and even through the skin. There growing, propagating, they excite inflammation, they rob the blood of some of its constituents to feed themselves. We have now shown that this "Germ Theory" is no fanciful device to cover up ignorance, but that it rests on a strong basis of common sense and reason.

Even if the microscope were not invented, and we knew nothing whatever of these microscopic beings, our reason alone, by mere induction, must lead us to a firm belief in a living and unseen contagium or parasite. And it did so, even two thousand years ago, for the Roman physicians gave credence to a parasitic theory of specific diseases.

But since then our beliefs have gained still stronger foundation. These germs are not imaginary. Even in our perfect health our bronchial tubes teem with different species of lower organisms. We need but to scrape our tongue with a knife blade, examine what is removed under the microscope, to find it swarming with the small slender rods of the leptothrix and alive with the rapidly-whirling spirillum, and the spores of oidium albicans. Any time you may examine bronchial secretions or the contents of the stomach, you will find them full of minute organisms of various species. The intestinal canal in every person is thronged with millions of them. The water we drink, the air we breathe are crowded with them. They are all parasites, every one, from the small dot-like microcosms to the branching, tree-like mildew. They cannot live without feeding upon organic matter. Put a piece of apple anywhere in a damp, warm place; it will soon be covered with a forest of mildew plants. Let a little milk stand in the same kind of a place; it will soon sour from the presence of the multitude of minute germs of fermentation.

But, it may be said, "Granted that these germs are everywhere present in countless myriads, which the microscope has revealed to us as true, have these minute creatures the power to produce such great results as the destruction of a whole community of human beings? Have they any power at all?" The answer is this: Pasteur has demonstrated that these bacteria are the chief cause of the alcoholic fermentation. It is known that they, in the shape of yeast, leaven our bread. They are more powerful than maggots in the destruction of carrion, for they are necessary to all decomposition; hence they may be said to bury our dead. A mighty power for good or evil cannot be denied to those animals, which, unseen by us, leaven the bread of millions of the people, make all the alcohol consumed in the world, give arrack to the Hindoos, make all the wine of France and Spain, all the beer of Germany and America, and virtually eat up millions of dead animals every year. Now, let us review a little: these diseases are infectious, contagious, epidemic — whatever the poison is, it grows and consequently is organic — these organisms were too small to be seen with the naked eye, hence at first their existence was but suspected; the microscope has discovered the presence of these germs everywhere in myriads; we have found them essential to decomposition and fermentation; later it was found, and proved beyond doubt, that they are absolutely the cause of diseases in numerous plants and lower animals. In 1838, for instance, the disease of the silk-worm, known as muscardine, was found to be due to fungi. But the most important discovery was that made by Pasteur, with regard to the silk-worm disease known as *pébrine*. For fifteen years the silk-worms had fallen victims in multitudes to the plague. The production of cocoons had fallen from 52,000,000 pounds, in 1853, to 8,000,000 in 1865. This was a dreadful blow to the silk industry in France. Everything in the pharmacopœia was put under contribution to prevent or cure the disease. It was found soon that the blood, all the tissues and even the eggs of the silk-worms, were full of minute vibratory corpuscles, which, indefinitely multiplying, caused the death of the insect. Pasteur experimented this way: He took a diseased worm and rubbed it up with water, spreading this out on mulberry leaves, with which he fed healthy worms. These last all became diseased from eating the corpusculous matter. After many such trials, he *proved* that it was this definite organism which caused the disease. Then he went to work to choose healthy eggs from the diseased worms, and gradually, slowly, but surely, the French, under his directions, have restored the old silk industry to its former proportions, and rooted out the fatal malady, *pébrine*. But to go from lower animals to man; it has been *conclusively* demonstrated that some human diseases are due to lower organisms. A fungus was found to be the cause of the skin disease known as favus or porrigo, and another to cause herpes tonsuraus, and still another to cause pityriasis versicolor. As to the specific fevers, it has been proved that in splenic fever of cattle the blood always contains bacteria, and that in this disease they are, at least, the cause. Soon after this, bacteria were found in most of the septic group, in pyæmia, septicæmia, puerperal fever, diphtheria, in vaccinia and small-pox. Then we had the weighty discovery of the spiral bacteria in the blood of patients with relapsing fever. Still later, the constant presence of bacteria was verified in cholera, endocarditis ulcerosa, scarlatina, and within a few months they have been discovered in tuberculosis and syphilis.

Still we are not satisfied; we must have positive, undeniable proofs, before we give the germ theory credit. The difficulty lies not in proving their presence in these diseases, for that has been sufficiently demonstrated, but in determining whether they are cause or effect. Do they really precede and give rise to the disease, or are they there because, when diseased, the body becomes a proper soil for them to grow in? What is the difference between those bacteria which are present in scarlet fever and those in small-pox? But here we stand on the border of that region which is all darkness, even to our microscopes. The bacteria of one septic disease seem precisely of the same form as those of any other. We have a single exception in relapsing fever where they are spiral. What are we to do? We cannot make our microscopes any better. Yet, though we cannot distinguish between bacteria of one fever and those of another, it is equally true that we see no difference between cells, which in one case become nerves, in another muscle, in another bone; indeed, I might say which in one case become men, in another case oxen or rats, for these cells, too, are apparently the same. But the truth is we must not think there is nothing more to see because our microscopes do not reveal more. It is probable that there are more startling things than we dream of to be seen with more perfect lenses. We know but little of the structure of the bacteria and their method of generation. Some suppose they proliferate by division, as do cells, but if they multiply as some other and larger vegetable parasites do, such as the common mildew, by throwing off of spores, then the bacteria being just visible with the microscope, their spores would be completely invisible. These spores, far out-numbering even the bacterian myriads, unseen to us, thronging the atmosphere everywhere, may be the carriers and originators of the disease, and only in their adult form become perceptible to us. This is beyond the power of the microscope. With a lens magnifying 500 times we can see quite plainly a bacterium $\frac{1}{3500}$ of an inch in length, and this is about the size of the bacillus of tubercle. With a lens magnifying 5,000 diameters there are still other germs to be seen, some reckoned by Beale to be $\frac{1}{100000}$ of an inch in diameter. The microscope cannot go much farther. But Tyndall has startled us with an instrument which reveals the presence of these motes when there is nothing to be seen with a magnifying power of 15,000 diameters. This instrument is a beam of electric light. With this he has searched the purest water and air and found in them these tiny particles far beyond the reach of the microscope, from which in time bacteria developed. It is proposed now to make a distinction between the words *germ* and *bacterium*. *Germ* is limited to these marvelously minute spores from which the bacteria develop. *Bacterium* is the adult form.

All sorts of experiments have been made with bacteria to separate them from the tissues, by filtration, freezing, by dialysis, in order then to test the infectious power of the tissue without bacteria, and the action of the bacteria themselves. For the same purpose chemicals and heat have been used. But the *germs* can outlive boiling, freezing, can pass through the strongest acids and filters—*they* can endure what is death to them in the adult bacterian form. Hence in liquids, boiled for hours, finally from the germs bacteria developed, and many thought this to be spontaneous generation, or development of life from inorganic, dead matter. Most of the septic poisons are peculiar to the human body alone; hence, experiments cannot be carried out upon lower animals.

It has been essayed to change the character of poisonous bacteria by cultivation in different fluids, and some success has followed these experiments in the hands of Pasteur and others. For instance, out of the bacteria of splenic fever, Pasteur claims to have made harmless bacteria by changing the chemical character of the fluids which contained them; and further, that by inoculation with these harmless bacteria but slight change is excited, and attacks of real splenic fever warded off. It is claimed that the small-pox bacteria are thus changed in passing through cattle, becoming quite harmless in the vaccine virus. None of these statements can be said as yet to have been satisfactorily demonstrated.

Another question to be answered is, suppose the bacteria are the cause of the disease, *how* do they cause it? It is thought by a very few that the bacteria themselves are poisonous; others believe they act by taking from the body the material necessary for its nourishment, that they use the oxygen of the blood, and still others think they give rise to a process in the blood similar to fermentation.

But, however they may act, the character of these infectious diseases, the necessity for a living and growing poison, the demonstrated presence of these bacteria or their germs everywhere, seem to me to make absolute truth of the statement that they are the cause of these diseases.

